# Overview of Long-Term Outcome in Adults with Systemic Right Ventricle and Transposition of the Great Arteries: A Review

**DOI:** 10.3390/diagnostics13132205

**Published:** 2023-06-28

**Authors:** Francesca Bevilacqua, Giulia Pasqualin, Paolo Ferrero, Angelo Micheletti, Diana Gabriela Negura, Angelo Fabio D’Aiello, Alessandro Giamberti, Massimo Chessa

**Affiliations:** 1 Adult Congenital Heart Disease Unit, Pediatric and Adult Congenital Heart Centre, IRCCS-Policlinico San Donato, 20132 Milano, Italy; giulia.pasqualin@grupposandonato.it (G.P.); paolo.ferrero@grupposandonto.it (P.F.); angelo.micheletti@grupposandonato.it (A.M.); dianagabriela.negura@grupposandonato.it (D.G.N.); angelofabio.daiello@grupposandonato.it (A.F.D.); massimo.chessa@grupposandonato.it (M.C.); 2 European Reference Network for Rare and Low Prevalence Complex Diseases of the Heart: ERN GUARD-Heart, 00165 Rome, Italy; alessandro.giamberti@grupposandonato.it; 3 Congenital Cardiac Surgery Unit, IRCCS-Policlinico San Donato, 20097 Milano, Italy; 4 Vita Salute San Raffaele University, 20132 Milano, Italy

**Keywords:** systemic right ventricle, transposition of the great artery, adult congenital heart disease, heart failure, sudden cardiac death

## Abstract

The population of patients with a systemic right ventricle (sRV) in biventricular circulation includes those who have undergone an atrial switch operation for destro-transposition of the great arteries (d-TGA) and those with congenitally corrected transposition of the great arteries (ccTGA). Despite the life expectancy of these patients is significantly increased, the long-term prognosis remains suboptimal due to late complications such as heart failure, arrhythmias, and premature death. These patients, therefore, need a close follow-up to early identify predictive factors of adverse outcomes and to implement all preventive therapeutic strategies. This review analyzes the late complications of adult patients with an sRV and TGA and clarifies which are risk factors for adverse prognosis and which are the therapeutic strategies that improve the long-term outcomes. For prognostic purposes, it is necessary to monitor sRV size and function, the tricuspid valve regurgitation, the functional class, the occurrence of syncope, the QRS duration, N-terminal pro B-type natriuretic peptide levels, and the development of arrhythmias. Furthermore, pregnancy should be discouraged in women with risk factors. Tricuspid valve replacement/repair, biventricular pacing, and implantable cardioverter defibrillator are the most important therapeutic strategies that have been shown, when used correctly, to improve long-term outcomes.

## 1. Introduction

In the context of biventricular circulation, the systemic right ventricle (sRV) is found in patients with dextro-transposition of the great arteries (d-TGA) palliated with an atrial switch operation (AtSO, commonly known as Mustard or Senning procedure) and in patients with congenitally corrected transposition of the great arteries (ccTGA or l(levo)-TGA).

d-TGA occurs in 1:3100 live births [1] and it is characterized by ventriculo-arterial discordance that results in separate pulmonary and systemic circulations (morphological right ventricle (RV) is connected to the aorta and morphological left ventricle (LV) is connected to the pulmonary artery). The AtSO consists of the creation of complex intra-atrial baffles to redirect systemic venous and pulmonary venous blood flow to the correct ventricle, leaving the RV in the systemic position [2,3].

While the ccTGA occurs in 1:33,000 live births [1] and it is characterized by both ventriculo-arterial and atrio-ventricular (AV) discordance (the right atrium is connected to the morphological LV which ejects blood into the pulmonary artery, whereas the left atrium is connected to the morphological RV, which ejects blood into to the aorta) [4]. This double discordance results in physiologically normal blood flow despite the RV being in a subaortic position.

Despite the AtSO being replaced by arterial switch operation in the early 1990s, patients palliated with AtSO and those with ccTGA currently represent an important cohort of adult patients with congenital heart disease (ACHD).

**Figure 1:**
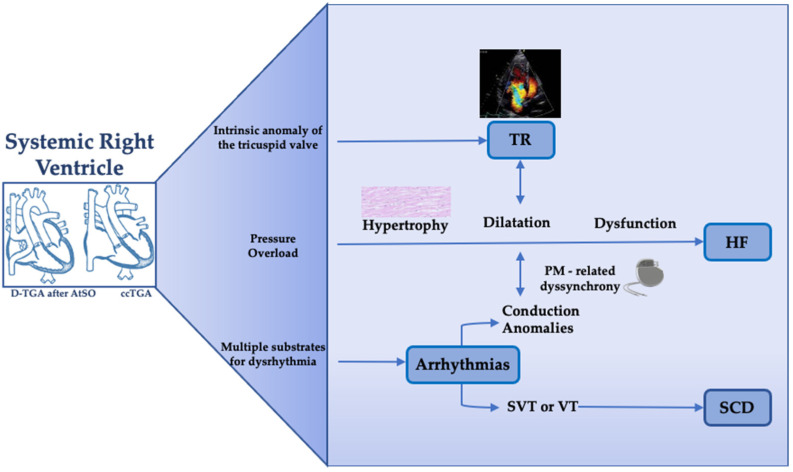
Long-term sequelae of the systemic right ventricle. The figure illustrates the major long-term complication of adult patients with a systemic right ventricle and the main underlying pathophysiological mechanisms. D-TGA, destro-transposition of the great arteries; ccTGA, congenitally corrected transposition of the great arteries; AtSO, atrial switch operation; TR, tricuspid regurgitation; HF, heart failure; PM, pacemaker; SVT, supraventricular tachycardia; VT, ventricular tachycardia; SCD, sudden cardiac death.

Indeed, in the last decades, advances in medical and surgical care have significantly improved the long-term survival of these patients, resulting in a higher incidence of long-term complications such as progressive sRV dysfunction (sRVd) and significant tricuspid regurgitation (TR) predisposing to heart failure (HF), arrhythmias and death [5,6,7,8] (Figure 1). Arrhythmias and sudden cardiac death (SCD) constitute most major events at young adult age, while HF and concurrently HF-related death are prevalent after the age of 40 years [9,10]. Prospective studies have demonstrated a cumulative survival of 60–89% after 30 years of follow-up in patients after AtSO [7,11,12,13] and a survival rate of 75% after 20 years of follow-up in ccTGA patients [14].

These late sequelae arise because the RV which normally supports the pulmonary low-pressure circulation when it is in a subaortic position undergoes a compensatory remodeling to be able to support the chronic pressure overload but, as a result of its intrinsic structural and contractile properties, the sRV does not assure long term performance [15,16].

Considering these data, adequate risk stratification of these patients is needed. This review analyzes the long-term complications of adult patients with an sRV and TGA and clarifies which are risk factors for adverse prognosis and which are the therapeutic strategies that improve the long-term outcomes.

## 2. Materials and Methods

Authors performed a search on PubMed for papers relating to “systemic right ventricle”, “outcome”, and “prognosis” until 1 March 2023.

Other keywords were “heart failure”, “sudden cardiac death” and “transposition of the great arteries”. Additionally, further studies were obtained through the references of some papers. The final reference list was developed based on originality and relevance to the broader scope of this review.

## 3. Heart Failure (HF)

Adult patients with an sRV and TGA have a significant risk to develop HF due to progressive sRVd, regurgitation of the systemic AV valve, and arrhythmias. By the age of 45, indeed, up to 65% of the patients with an sRV present symptomatic HF [17,18].

Since HF is associated with an adverse outcome, prevention and early treatment of HF are crucial in this group of patients [19].

Therapies to treat HF in this population include drugs, catheter-based interventions, pacemakers, cardiac resynchronization therapy (CRT), and surgical interventions to address tricuspid valve regurgitation and associated cardiac defects. Mechanical assist devices and heart transplants remain the intervention for end-stage HF.

### 3.1. Systemic Right Ventricle Dysfunction (sRVd)

The sRV is prone to develop systolic dysfunction over time due to its inability to sustain systemic circulation in the long run [20]. Among patients with an sRV after AtSO, asymptomatic sRVd is already present within the 3rd decade of life, while more than 50% of patients in their 4th or 5th decade of life present clear signs of HF [10,15]. Equally, the prevalence of sRVd in cc-TGA patients ranging from 55% to 80% across the studies, and most patients manifest symptoms related to HF during the 4th or 5th decade of life [17,21,22,23,24].

The sRV failure is mainly due to its inability to adapt to high pressures, leading to ventricular hypertrophy, and later dilatation, dysfunction, and finally HF with significant long-term morbidity and mortality [17,25,26,27,28,29].

Among factors that contribute to sRVd, there are:

- The coronary insufficiency due to single coronary perfusion of sRV (right coronary artery) that could be responsible for myocardial fibrosis development [30,31,32]; indeed, both focal and myocardial diffuse fibrosis, as assessed by cardiac magnetic resonance (CMR) delayed enhancement imaging, have been correlated with sRVd and adverse long-term outcome [31,33,34];
- Progressive TR, usually secondary to annular enlargement and/or to intrinsic anomaly of the tricuspid valve (frequent features in ccTGA patients);
- Arrhythmias, especially the conduction disorders and AV block (they are found in up to 50% of patients with ccTGA [15]) as they require pacemakers; indeed, chronic LV pacing may induce pacemaker-related dyssynchrony [27,35], worsening sRV dilation and failure, as well as TR [14,36];
- Associated cardiac lesions (up to 80% of patients with CCTGA have them, such as ventricular septal defect, pulmonary stenosis and Ebstein anomaly of tricuspid valve) [36,37,38,39]; sRV function is decreased in patients with ccTGA undergoing physiologic repair (compared with those without previous surgery) [40,41] possibly due to the presence of hemodynamically significant concomitant lesions before surgery and the inflammatory response associated with cardiopulmonary bypass [42];
- Ventriculo-ventricular interaction driven by LV systolic dysfunction [22];
- Residual lesions, especially in d-TGA patients after palliation [43].

Since the sRVd is one of the main contributors to mortality and morbidity in this population [36], its early recognition and relatively appropriate treatment has significant clinical relevance [44,45].

CMR is useful in identifying subclinical sRVd and it is considered the gold standard to measure volumes of the sRV with strong predictability of adverse outcomes [46,47,48]. A recent study demonstrated that CMR measurements of sRV volumes were strong predictors of death and end-stage HF and, for patients after AtSO, were superior to systemic ventricular ejection fraction for predicting adverse events: an RV end-diastolic volume indexed (RVEDVI) cut-points of 130 mL/m^2^ for survival analysis was significantly associated with survival in patients after AtSO as well as in patients with ccTGA [47].

Global circumferential strain has also been proposed for the early detection of sRVd [49] but the current literature is unclear regarding the prognostic value of strain analysis in sRV patients [21,50,51]

Moreover, recent studies revealed a correlation of left ventricular outflow tract obstruction (LVOTO) with sRV function, suggesting a positive effect of the LVOTO on hemodynamic status and thus a potential protective effect on sRV systolic in the long-term [52,53].

Despite several therapies that have been proposed for the treatment of sRVd, their efficacy has not been consistent across different studies [23,40,54]. Since there are no validated therapies to reverse sRVd once it occurs, the aim of care should, therefore, be to prevent the occurrence and progression of sRVd.

### 3.2. Tricuspid Valve Regurgitation (TR)

TR is one of the most common hemodynamic lesions in patients with an sRV (especially in patients with ccTGA). Indeed, chronic volume overload from TR, coupled with systemic afterload, results in a high prevalence of sRVd and HF in patients with an sRV [17,23,36,50,55,56]. Around 40–57% of ccTGA patients have moderate to severe TR [17]; whereas, severe TR is reported in 8% of patients with TGA post-AtSO [10].

TR is due to both the intrinsic anomaly of the tricuspid valve (dysplastic valves such as Ebsteinoid leaflets commonly found in cc-TGA) and the consequence of sRV dilatation and ensuing annular enlargement. As mentioned above, the sRV is only poorly suited to work as a high-pressure ventricle, predisposing the patients to dilatation of sRV and secondary TR [18]. The mechanism by which TR increases ventricular and annular dilation which, in turn, worsens TR is a vicious circle [28,57,58]. There is a point where further RV dilation increases an individual’s risk for a cardiac event irrespective of the degree of TR [47]. Progressive RV enlargement may also alter ventricular function by impairing contractility, a phenomenon that is only partially attenuated by RV hypertrophy from exposures to chronic overload [59].

TR has a significant clinical impact on these patients and is an independent predictor of adverse outcomes [8,17,60,61]. Indeed, TR was found to be the strongest risk factor of mortality both in post-AtSO and ccTGA patients [60,62].

Among patients with ccTGA, the survival rate, after a follow-up of 20 years, appears to be strongly influenced by TR severity (passing from 49% in patients with moderate or severe TR to 93% in those without) [60].

The role of the TR as a potential therapeutic target in patients with an sRV and TGA has been well recognized. Tricuspid valve replacement (TVR) is an effective therapy for the management of TR in this group of patients, and it is associated with the improvement of sRV systolic function, especially when performed before the onset of significant sRVd [22,23,63,64]. This has important clinical implications in the management of patients where the TR is not considered significant enough to recommend surgical replacement: in these patients, the RV systolic function should be closely monitored, and these patients should undergo TVR if there is a reduction of RV systolic function without waiting for the TR to become severe.

Unlike d-TGA post-AtSO (where TR is generally secondary to annular dilatation and thus TVR is not always justified), the main prognostic intervention in patients with ccTGA is aimed at improvement in TR [8].

The Mayo Clinic reported outcomes for tricuspid interventions in ccTGA patients: patients who underwent surgery before the RV ejection fraction deteriorated to less than 40% maintained preserved RV function long-term [23].

Currently, recommendations for surgical intervention on the tricuspid valve are limited to symptomatic patients or asymptomatic patients with severe TR and progressive sRV dilation. In both indications, patients must have RV ejection fraction > 40% [6].

### 3.3. Arrhythmias

Among the most common complications for all patients with an sRV, there are arrhythmias, both brady- and tachyarrhythmias.

AV node and sinoatrial node dysfunction are two of the most common reasons to require permanent pacing. Patients with cc-TGA have an annual risk of developing a de novo AV-block of ~2%, due to the abnormal disposition of the conduction system [6,65,66]. In patients with d-TGA after AtSO, the most common arrhythmia requiring pacing is a sinus node dysfunction (prevalence ≤ 60% at 20 years of follow-up) [67] caused by injury during surgery [61,68]. The loss of sinus rhythm is significantly associated with an increased risk of adverse cardiac outcomes [50] in this group of patients.

In the long run, chronic subpulmonary ventricular pacing lead to pacing-induced dyssynchrony, which further worsens the sRVd and can cause HF [37,69].

The chronic pacing-related dyssynchrony may be prevented by biventricular pacing, shown to preserve or restore sRV function [37,54,70], although, the data supporting the use of CRT are rather limited and often conflicting [22,37,54,71].

Small studies on CRT in sRV patients suggest improvement in sRV systolic and diastolic function and in functional class [71] but no benefit on TR; therefore, concomitant tricuspid valve surgery for patients with ccTGA may be required [70]. Comparatively, patients with an sRVd who undergo TV surgery are prone to develop AV-conduction disorders requiring chronic ventricular pacing [72]. Therefore, concurrent epicardial sRV lead implantation at the time of TV surgery and timely initiation of CRT is to be considered [72].

Moreover, careful patient evaluation and selection are essential in achieving successful CRT [69,70,73]. The reliable predictors of CRT response in patients with an sRV are CRT implantation with the need for ventricular pacing (vs for wide QRS complex), female sex, narrower paced QRS, higher baseline systemic ejection fraction of sRV and epicardial or hybrid CRT devices [71,74]. Furthermore, the lead placement on the RV free wall was associated with better outcomes compared with lead placement on the RV outflow tract [75].

Currently, CRT devices are recommended:

1. in patients with a sRV and systemic ejection fraction ≤ 35% and QRS duration ≥ 150 ms (spontaneous or paced) as HF therapy.
2. or preserved systemic ejection fraction undergoing new device placement or replacement with an anticipated requirement for significant (>40%) ventricular pacing, considering data that suggested that it may lead to a better preservation of RV systolic function than LV pacing alone [6,37,65,67,76,77]
3. in patients with functional class IV and severe ventricular dysfunction as a bridge to mechanical assist device therapy or heart transplantation [6,65].

In adults with an sRV, approximately 15% of patients would be candidates for CRT using current indication criteria.

These data show the potential of CRT as a therapeutic candidate in properly selected patients.

### 3.4. Functional Class and Exercise Capacity

Most of the patients with an sRV and TGA are classified as New York Heart Association (NYHA) functional class I and II, are well adapted, and report no symptoms despite the presence of significant sRVd and an objectively reduced exercise capacity on cardiopulmonary exercise testing (CPET) [78,79,80,81]. This reduced exercise tolerance is mainly related to chronotropic incompetence [82] and a limited capacity to increase stroke volume during exercise [83]. Although it’s a subjective aspect, higher NYHA class is significantly associated with adverse outcomes (such as death, HF, and arrhythmia) in patients with a sRV [62,84].

CPET might help to determine functional capacity and has a predictive value for adverse outcomes in adults with CHD [85]. Among CPET parameters that showed a strong association with adverse cardiac events, there are the indexed oxygen uptake efficiency slope (OUES) [82], VO2, and VE/VCO2 [86]

These results have the potential to become a useful clinical tool for surveillance of this high-risk patient population. Training may improve exercise capacity, and patients not considered to be at significant risk for arrhythmias or SCD during exercise should be encouraged to regular physical activity.

### 3.5. Blood Biomarkers

N-terminal pro-B-type natriuretic peptide (NT-proBNP) is the most used and studied blood biomarker for risk stratification in patients with CHD [87] and is one of the few blood biomarkers that has been studied in patients with an sRV. NT-proBNP, as an indicator of hemodynamic burden, has been shown in many studies to be a surrogate marker for mortality and HF in patients with an sRV [50,80,81,88]. Prior studies have reported higher NT-proBNP levels in association with higher NYHA functional class, more severe TR, more severely impaired ventricular function, QRS prolongation, and age [89,90,91]. Even in mildly symptomatic patients, NT-proBNP levels provide independent prognostic information for long-term outcomes regarding all causes of adverse cardiac events and, in particular, HF, transplantation, and death [81].

NT-proBNP levels > 1000 mg/mL have been associated with a high risk of death in patients after AtSO [88].

Furthermore, GDF-15 is a blood biomarker secreted in response to multiple processes, including hypoxia and inflammation and it appears to be better as a predictor for adverse long-term outcomes than NT-proBNP in patients with sRV [50].

In a recent study, microRNA-183-3p and highly sensitive troponin T were found to be independent predictors of worsening HF in this group of patients [92].

The data available to date show that the use of biomarkers has an important predictive value for adverse outcomes in patients with an sRV and TGA [93].

### 3.6. Medical Therapy

To date, the role of medical therapy in the treatment of HF in these patients remains unclear. A recent meta-analysis concluded that, due to the small sample size of the available studies, there is no strong evidence for the effectiveness of medical therapy with beta-blockers, angiotensin-converting enzyme inhibitors, angiotensin receptor blockers, or aldosterone antagonists in patients with sRVd and HF [94].

However, a randomized controlled trial of the angiotensin II receptor blocker Valsartan in adult patients with a sRV failed to show an improved survival at longer-term follow-up but was associated with decreased risk of cardiac events (such as arrhythmias, worsening of HF or TVR) in symptomatic patients [95].

Regarding the use of angiotensin receptor-neprilysin inhibitor (ARNI), there is preliminary evidence showing potential benefit in sRV patients; this treatment seems to be associated with significant improvements in NT-proBNP levels, physical activity, quality of life, and sRV function in a cohort of adult patients with sRVd (ejection fraction ≤ 35%) who were treated for six months [96].

In conclusion, to date, in contrast to patients with LV-HF, there is no guideline-directed medical therapy with a proven effect on morbidity or mortality in sRVd.

### 3.7. Surgical and Catheter Interventions

Among the management options proposed to improve or prevent sRVd, there are surgical and catheter interventions such as pulmonary artery banding (PAB) and correction of late postoperative complications (especially in patients post-AtSO). The role of tricuspid valve surgery has already been discussed.

The goal of PAB may be palliative or curative, in the latter case with the idea of reconditioning the LV before performing an anatomic repair. PAB could reduce TR and improve RV function by increasing subpulmonary LV pressure which reduces the septal shift and increases leaflet coaptation [97,98]. However, prolonged PAB may expose older children and adults to the risk of diastolic dysfunction, potentially compromising their outcome [99]. Outcomes of LV retraining with staged, serial PAB, followed by anatomic repair beyond childhood have been linked to a high risk of LV dysfunction, impossibility to proceed to anatomic repair, and an increase in perioperative mortality when an anatomic repair takes place [100].

Complications after AtSO such as systemic baffle obstruction or leak account for most reinterventions, whereas pulmonary baffle obstruction is less common but may cause pulmonary hypertension [28,101]. These late sequelae are associated with an adverse outcome [28,33,102], hence the importance of timely treatment.

### 3.8. Mechanical Circulatory Support and Heart Transplantation

Patients with an sRV in end-stage HF should be referred to a transplant center for advanced mechanical circulatory support devices or heart transplantation (HT).

Determining the optimal timing of HT in these patients is difficult because most of the patients seem to be clinically stable for a long time and their hemodynamic deterioration may be rapid and unexpected. Moreover, outcomes after HT remain often unsatisfactory with high short-term mortality. However, this high early risk is counterbalanced by better long-term survival, superior to other groups [103].

Since transplant waiting lists are often long and there is high mortality on the waiting list, it seems prudent to refer these patients to an advanced center to consider mechanical circulatory support devices before other organ failure and pulmonary arterial hypertension occur [103,104].

Currently, the use of mechanical circulatory support devices for the failing sRV is rather limited [26,105,106]. A ventricle assistant device (VAD) can be used as a bridge to transplantation or as a destination therapy, the latter limited to selected patients [107]. Despite anatomical challenges with VAD implantation in the sRV [108], these devices reduce pulmonary vascular resistance as a bridge to the transplant [109]. When following this strategy, additional treatment of more than moderate TR at the time of VAD implantation may well be considered, aiming for a better long-term outcome [110]. However, the number of patients treated in this way is still limited and patient selection and fighting the burden of complications related to VAD therapy remain a considerable challenge.

### 3.9. Effect of Pregnancy

Although the pregnancy is usually well tolerated for most women with sRV, the risk for adverse cardiac events is not insignificant since sRV may be inadequate to respond to the physiologically increased workload which occurs in pregnancy.

Furthermore, the cardiac complications that occur during pregnancy, including HF, arrhythmias, thromboembolic events, worsening of sRV function, and TR, may also persist after delivery with possible long-term effects [111]. Arrhythmias and HF are the most common complications, ranging respectively from seven to 22% and from seven to 21% of events across the studies [112,113,114,115]. Arrhythmias occur more likely in the second trimester, whereas HF is more frequent in the third trimester or in the early postpartum period when volume overload reaches the maximum level [114,116].

Pre-existing moderate sRVd, moderate to severe TR, and previous history of arrhythmias are linked with a high rate of serious complications [112,114,116], but irreversible sRVd and functional deterioration may occur during and after pregnancy even in women without preexisting pathological features [113,115,117].

In conclusion, sRV carries a high risk of maternal complications, regardless of clinical status and hemodynamic stability at the time of conception. According to current guidelines, in patients with more than moderate impairment of RV function or greater than moderate TR, pregnancy should be discouraged [111].

## 4. Sudden Cardiac Death (SCD)

Patients with a sRV experience decreased survival compared to that of the general population. Indeed, patients after AtSO and patients with ccTGA are among ACHD patients at the highest risk for SCD, with reported rates ranging from 2.4 to 3.7 and from 1.8 to 25.0 per 1000 patient-years, respectively [118].

**Figure 2:**
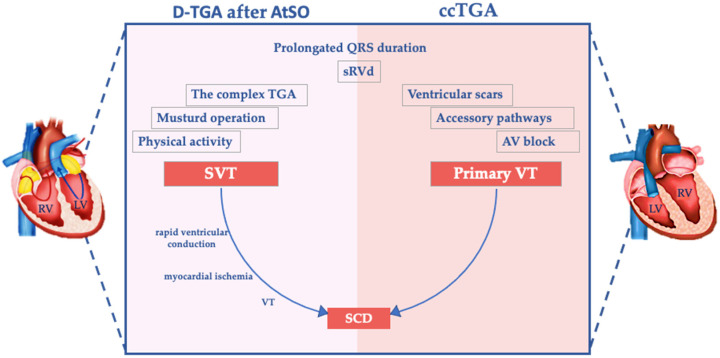
Potential substrates for sudden cardiac death in patients with an sRV and TGA. The figure describes the potential substrates and triggers for SCD in patients with TGA after AtSO and in patients with ccTGA. sRVd, systemic right ventricle dysfunction; AV, atrioventricular.

D-TGA and ccTGA share some, but not all, potential factors associated with a high risk of SCD [119] (Figure 2).

Among patients after AtSO, the most common cause of death is SCD followed by HF [120]. Notably, SCD is the most common cause in adolescence and HF is the main cause in adulthood [120,121].

The main triggers associated with an increased risk of SCD in patients after AtSO are listed below.

1. Supraventricular Tachycardia (SVT) showed a significant impact on the risk of SCD [62]. The incidence of SVT increases with aging and affects up to a third of these patients [19,122]. In the presence of rapid ventricular conduction to the sRV with systolic and/or diastolic dysfunction, this may result in low cardiac output, which may induce myocardial ischemia, potentially leading to ventricular tachycardia (VT) and exposing the patients at risk for SCD [62,123]. In addition, primary VT may also occur, most often in association with sRVd. Limited data suggest that the use of beta-blockers provides some protection [124] and timely ablation of SVT could theoretically avoid events.
2. The Mustard procedure is a significant risk factor for SCD [9]. A possible explanation for this could be due to the differences in the surgical techniques between the Mustard and Senning operations. Indeed, the Mustard method brought about more complications compared with the Senning technique, such as baffle obstruction, sinus rhythm disturbances, and SVT.
3. The complex TGA compared with simple TGA is a significant risk factor for all-cause mortality, including SCD [9,11].
4. Exercise is a well-known trigger of SCD. Indeed, SCD in D-TGA patients happened during physical activity in about 80% of the patients [123,125], probably because SVT can occur during exercise.
5. Prolongated QRS duration, as an expression of intra-myocardial fibrosis that putatively develops as a maladaptive response in the failing sRV [34], increases the risk of ventricular arrhythmia and/or SCD both in patients after AtSO and also in patients with ccTGA [5,47,119,126].
6. sRVd, NYHA class ≥ III/HF hospitalization, and least moderate TR are long-term all-cause mortality risk factors in patients after AtSO [62].

Similarly, the most common causes of death for patients with ccTGA are HF and SCD. The main factors of increased SCD risk in patients with ccTGA are listed below.

1. Primary ventricular tachycardia occurs in about 20% of the patients with ccTGA, especially in those with sRVd [53,126]. This prevalence, which is higher compared to patients after AtSO, may be explained by multiple substrates for dysrhythmia ventricular scars in patients undergoing a physiologic repair strategy (such as the presence of or a possible association with accessory pathways in patients with Ebstein-like valve).
2. SRVd is a well-known risk factor for SCD, in particular in patients with sRV EF < 35% [126].
3. AV block may increase the risk of SCD. Indeed, the fibrosis of the proximal non-bifurcating His bundle can constitute an underlying arrhythmogenic substrate [127].

In addition, the pacing is strongly associated with the risk of SCD [119]. Indeed, in the setting of AV block, there is a strong association between subpulmonary ventricular pacing with pacemaker-induced ventricular dyssynchrony and dysfunction that exposes patients to high risk of SCD, hence the need, as already mentioned, to consider CTR as primary therapy in this setting [37].

Moreover, given the high incidence of arrhythmias in this population and their impact on prognosis, it is essential to recognize and treat them as soon as possible. Wearable devices that allow subclinical and early diagnosis of rhythm abnormalities can play a significant role in arrhythmias detection and in supporting treatments in the future.

### Implantable Cardioverter Defibrillator (ICD)

Despite the well-known risk of SCD in patients with an sRV and TGA, risk stratification for implantable cardioverter-defibrillator (ICD) for primary prevention of SCD remains unclear [128,129]. Furthermore, not all SCDs are due to shockable rhythms (particularly in the setting of advanced HF) [118] and appropriate ICD therapy overestimates the risk for SCD by two- to three-fold [130].

According to current guidelines, ICD for primary prevention may be considered for adults patients with an sRV with an ejection fraction < 35% in the presence of additional risk factors such as NYHA functional class II-III, syncope, documents as non-sustained ventricular tachycardia (or complex ventricular arrhythmias), QRS ≥ 140 ms, or severe TR [67,131].

ICD for secondary prevention represents the first-line therapy, whereas pharmacotherapy may be added to reduce the burden of implantable cardioverter defibrillator shocks.

## 5. Risk Assessment Models

The risk stratification of patients with an sRV has considerable clinical relevance. A recent study showed a mortality rate of 25% at 10-year follow-up, in adult patients with an sRV and TGA, with 52% of patients reaching the composite endpoint of mortality, progressive VAD or HF implantation requiring CRT [72]. Determining predictors of worse outcomes in this group of patients may help to improve long-term prognosis.

Recently, a clinical risk score was proposed to predict major clinical events in patients after AtSO [10]. This model stratifies patients into low, intermediate, and high-risk groups for event-free survival based on six variables: age > 30 years, repair at >one year, previous ventricular arrhythmias, at least moderate RV and mild LV dysfunction, and severe TR.

A large multicenter study of adult patients post AtSO [121] identifies as factors associated with a composite outcome of death, HT, and mechanical circulatory support during a mean follow-up of 8.9 years, the history of complex anatomy, prolonged QRS, HF admission, severe sRVd, and ventricular arrhythmias. These variables were used to create a prediction tool for five-year survival in the adult post-AtSO for d-TGA [121].

An additional risk stratification model has recently been proposed to estimate the risk for ventricular arrhythmias and SCD at five years in patients with sRV [119]. The six variables included in this model are age at baseline, history of HF, syncope, QRS duration, severe sRVd, and at least moderate left ventricular outflow tract (LVOT) obstruction > 36 mmHg [119]. Although LVOT obstruction was found to be associated with a lower risk of developing HF or an additional protective factor [53,132], as mentioned above, the resulting LV hypertrophy may contribute to the ventricular arrhythmia substrate.

Furthermore, genetic predisposition may play a role in sRVd and subsequent clinical events. Common single-nucleotide polymorphisms may be implicated in the heterogeneous clinical course of TGA after AtSO [133]. The addition of genetic information and, in general, the multi-omics approach can improve risk prediction over the use of a clinical risk model alone.

In conclusion, risk scores are a useful tool to help clinicians identify high-risk patients who could benefit from a more aggressive treatment approach.

## 6. Conclusions

Adult patients with an sRV comprise a distinctly clinically challenging group of patients with increased morbidity and mortality. It is known that a right ventricle in the systemic position is prone to maladaptive remodeling, so these patients are at high risk for HF, arrhythmias, and death in adult life.

Currently, the management of patients with an sRV is hampered by the lack of evidence-based recommendations because randomized clinical trials are difficult to perform due to the limited population size. However, an individual approach and careful risk stratification as well as early therapeutic strategies are crucial to improve long-term outcomes.

## Data Availability

Not applicable.

## References

[1] Iriart X., Roubertie F., Jalal Z., Thambo J.-B. (2016). Quantification of Systemic Right Ventricle by Echocardiography. Arch. Cardiovasc. Dis..

[2] Mustard W.T. (1964). Successful Two-Stage Correction Of Transposition Of The Great Vessels. Surgery.

[3] Senning A. (1959). Surgical Correction of Transposition of the Great Vessels. Surgery.

[4] Kutty S., Danford D.A., Diller G.-P., Tutarel O. (2018). Contemporary Management and Outcomes in Congenitally Corrected Transposition of the Great Arteries. Heart.

[5] Schwerzmann M., Salehian O., Harris L., Siu S.C., Williams W.G., Webb G.D., Colman J.M., Redington A., Silversides C.K. (2009). Ventricular Arrhythmias and Sudden Death in Adults after a Mustard Operation for Transposition of the Great Arteries. Eur. Heart J..

[6] Baumgartner H., De Backer J., Babu-Narayan S.V., Budts W., Chessa M., Diller G.-P., Lung B., Kluin J., Lang I.M., Meijboom F. (2021). 2020 ESC Guidelines for the Management of Adult Congenital Heart Disease. Eur. Heart J..

[7] Vejlstrup N., Sørensen K., Mattsson E., Thilén U., Kvidal P., Johansson B., Iversen K., Søndergaard L., Dellborg M., Eriksson P. (2015). Long-Term Outcome of Mustard/Senning Correction for Transposition of the Great Arteries in Sweden and Denmark. Circulation.

[8] Warnes C.A. (2006). Transposition of the Great Arteries. Circulation.

[9] Venkatesh P., Evans A.T., Maw A.M., Pashun R.A., Patel A., Kim L., Feldman D., Minutello R., Wong S.C., Stribling J.C. (2019). Predictors of Late Mortality in D-Transposition of the Great Arteries after Atrial Switch Repair: Systematic Review and Meta-Analysis. J. Am. Heart Assoc..

[10] Woudstra O.I., Zandstra T.E., Vogel R.F., van Dijk A.P.J., Vliegen H.W., Kiès P., Jongbloed M.R.M., Egorova A.D., Doevendans P.A.F.M., Konings T.C. (2021). Clinical Course Long after Atrial Switch: A Novel Risk Score for Major Clinical Events. J. Am. Heart Assoc..

[11] Antonová P., Rohn V., Chaloupecky V., Simkova I., Kaldararova M., Zeman J., Popelova J., Havova M., Janousek J. (2022). Predictors of Mortality after Atrial Correction of Transposition of the Great Arteries. Heart.

[12] Cuypers J.A.A.E., Eindhoven J.A., Slager M.A., Opi P., Utens E.M.W.J., Helbing W.A., Witsenburg M., van den Bosch A.E., Ouhlous M., van Domburg R.T. (2014). The Natural and Unnatural History of the Mustard Procedure: Long-Term Outcome up to 40 Years. Eur. Heart J..

[13] Moons P., Gewillig M., Sluysmans T., Verhaaren H., Viart P., Massin M., Suys B., Budts W., Pasquet A., De Wolf D. (2004). Long Term Outcome up to 30 Years after the Mustard or Senning Operation: A Nationwide Multicentre Study in Belgium. Heart.

[14] Rutledge J.M., Nihill M.R., Fraser C.D., Smith O.E., McMahon C.J., Bezold L.I. (2002). Outcome of 121 Patients with Congenitally Corrected Transpositionof the Great Arteries. Pediatr. Cardiol..

[15] Andrade L., Carazo M., Wu F., Kim Y., Wilson W. (2020). Mechanisms for Heart Failure in Systemic Right Ventricle. Heart Fail. Rev..

[16] Friedberg M.K., Redington A.N. (2014). Right versus Left Ventricular Failure: Differences, Similarities, and Interactions. Circulation.

[17] Graham T.P., Bernard Y.D., Mellen B.G., Celermajer D., Baumgartner H., Cetta F., Connolly H.M., Davidson W.R., Dellborg M., Foster E. (2000). Long-Term Outcome in Congenitally Corrected Transposition of the Great Arteries: A Multi-Institutional Study. J. Am. Coll. Cardiol..

[18] Piran S., Veldtman G., Siu S., Webb G.D., Liu P.P. (2002). Heart Failure and Ventricular Dysfunction in Patients with Single or Systemic Right Ventricles. Circulation.

[19] Murphy D.J. (2005). Transposition of the Great Arteries: Long-Term Outcome and Current Management. Curr. Cardiol. Rep..

[20] Brida M., Diller G.-P., Gatzoulis M.A. (2018). Response by Brida et al. to Letter Regarding Article, “Systemic Right Ventricle in Adults with Congenital Heart Disease: Anatomic and Phenotypic Spectrum and Current Approach to Management”. Circulation.

[21] Diller G.-P., Radojevic J., Kempny A., Alonso-Gonzalez R., Emmanouil L., Orwat S., Swan L., Uebing A., Li W., Dimopoulos K. (2012). Systemic Right Ventricular Longitudinal Strain Is Reduced in Adults with Transposition of the Great Arteries, Relates to Subpulmonary Ventricular Function, and Predicts Adverse Clinical Outcome. Am. Heart J..

[22] Egbe A.C., Miranda W.R., Jain C.C., Connolly H.M. (2022). Prognostic Implications of Progressive Systemic Ventricular Dysfunction in Congenitally Corrected Transposition of Great Arteries. JACC Cardiovasc. Imaging.

[23] Mongeon F.-P., Connolly H.M., Dearani J.A., Li Z., Warnes C.A. (2011). Congenitally Corrected Transposition of the Great Arteries Ventricular Function at the Time of Systemic Atrioventricular Valve Replacement Predicts Long-Term Ventricular Function. J. Am. Coll. Cardiol..

[24] Liu R., Pang K., Li S., Zhang B., Rui L., Lin Y., Wang C., Ma K. (2021). The Fate of Congenitally Corrected Transposition of the Great Arteries Unoperated Before Adulthood. Ann. Thorac. Surg..

[25] Puley G., Siu S., Connelly M., Harrison D., Webb G., Williams W.G., Harris L. (1999). Arrhythmia and Survival in Patients >18 Years of Age after the Mustard Procedure for Complete Transposition of the Great Arteries. Am. J. Cardiol..

[26] Menachem J.N., Schlendorf K.H., Mazurek J.A., Bichell D.P., Brinkley D.M., Frischhertz B.P., Mettler B.A., Shah A.S., Zalawadiya S., Book W. (2020). Advanced Heart Failure in Adults with Congenital Heart Disease. JACC Heart Fail..

[27] Norozi K., Wessel A., Alpers V., Arnhold J.O., Geyer S., Zoege M., Buchhorn R. (2006). Incidence and Risk Distribution of Heart Failure in Adolescents and Adults with Congenital Heart Disease after Cardiac Surgery. Am. J. Cardiol..

[28] Rooshesselink J. (2004). Decline in Ventricular Function and Clinical Condition after Mustard Repair for Transposition of the Great Arteries (a Prospective Study of 22?29 Years)*1. Eur. Heart J..

[29] Winter M.M., Bouma B.J., Groenink M., Konings T.C., Tijssen J.G.P., van Veldhuisen D.J., Mulder B.J.M. (2009). Latest Insights in Therapeutic Options for Systemic Right Ventricular Failure: A Comparison with Left Ventricular Failure. Heart.

[30] Al-Wakeel-Marquard N., Ferreira da Silva T., Berger F., Kuehne T., Messroghli D.R. (2022). Myocardial Extracellular Volume Is a Non-Invasive Tissue Marker of Heart Failure in Patients with Transposition of the Great Arteries and Systemic Right Ventricle. Front. Pediatr..

[31] Babu-Narayan S.V., Goktekin O., Moon J.C., Broberg C.S., Pantely G.A., Pennell D.J., Gatzoulis M.A., Kilner P.J. (2005). Late Gadolinium Enhancement Cardiovascular Magnetic Resonance of the Systemic Right Ventricle in Adults with Previous Atrial Redirection Surgery for Transposition of the Great Arteries. Circulation.

[32] Hornung T.S., Kilner P.J., Davlouros P.A., Grothues F., Li W., Gatzoulis M.A. (2002). Excessive Right Ventricular Hypertrophic Response in Adults with the Mustard Procedure for Transposition of the Great Arteries. Am. J. Cardiol..

[33] Rydman R., Gatzoulis M.A., Ho S.Y., Ernst S., Swan L., Li W., Wong T., Sheppard M., McCarthy K.P., Roughton M. (2015). Systemic Right Ventricular Fibrosis Detected by Cardiovascular Magnetic Resonance Is Associated with Clinical Outcome, Mainly New-Onset Atrial Arrhythmia, in Patients after Atrial Redirection Surgery for Transposition of the Great Arteries. Circ. Cardiovasc. Imaging.

[34] Ladouceur M., Baron S., Nivet-Antoine V., Maruani G., Soulat G., Pereira H., Blanchard A., Boutouyrie P., Paul J.L., Mousseaux E. (2018). Role of Myocardial Collagen Degradation and Fibrosis in Right Ventricle Dysfunction in Transposition of the Great Arteries after Atrial Switch. Int. J. Cardiol..

[35] Horovitz A., De Guillebon M., van Geldorp I.E., Bordachar P., Roubertie F., Iriart X., Douard H., Haissaguerre M., Thambo J.-B. (2012). Effects of Nonsystemic Ventricular Pacing in Patients with Transposition of the Great Arteries and Atrial Redirection. J. Cardiovasc. Electrophysiol..

[36] Filippov A.A., Del Nido P.J., Vasilyev N.V. (2016). Management of Systemic Right Ventricular Failure in Patients with Congenitally Corrected Transposition of the Great Arteries. Circulation.

[37] Hofferberth S.C., Alexander M.E., Mah D.Y., Bautista-Hernandez V., del Nido P.J., Fynn-Thompson F. (2016). Impact of Pacing on Systemic Ventricular Function in L-Transposition of the Great Arteries. J. Thorac. Cardiovasc. Surg..

[38] Voskuil M., Hazekamp M.G., Kroft L.J.M., Lubbers W.J., Ottenkamp J., van der Wall E.E., Zwinderman K.H., Mulder B.J.M. (1999). Postsurgical Course of Patients with Congenitally Corrected Transposition of the Great Arteries. Am. J. Cardiol..

[39] Brida M., Diller G.-P., Gatzoulis M.A. (2018). Systemic Right Ventricle in Adults with Congenital Heart Disease: Anatomic and Phenotypic Spectrum and Current Approach to Management. Circulation.

[40] Barrios P.A., Zia A., Pettersson G., Najm H.K., Rajeswaran J., Bhimani S., Karamlou T. (2021). Members of the ccTGA Working Group Outcomes of Treatment Pathways in 240 Patients with Congenitally Corrected Transposition of Great Arteries. J. Thorac. Cardiovasc. Surg..

[41] Wilson H.C., Lu J.C., Yu S., Lowery R., Mahani M.G., Agarwal P.P., Dorfman A.L. (2022). Ventricular Function in Physiologically Repaired and Unrepaired Congenitally Corrected Transposition of the Great Arteries. Am. J. Cardiol..

[42] Riesenkampff E., Luining W., Seed M., Chungsomprasong P., Manlhiot C., Elders B., McCrindle B.W., Yoo S.-J., Grosse-Wortmann L. (2016). Increased Left Ventricular Myocardial Extracellular Volume Is Associated with Longer Cardiopulmonary Bypass Times, Biventricular Enlargement and Reduced Exercise Tolerance in Children after Repair of Tetralogy of Fallot. J. Cardiovasc. Magn. Reson..

[43] Derrick G.P., Narang I., White P.A., Kelleher A., Bush A., Penny D.J., Redington A.N. (2000). Failure of Stroke Volume Augmentation during Exercise and Dobutamine Stress Is Unrelated to Load-Independent Indexes of Right Ventricular Performance after the Mustard Operation. Circulation.

[44] Zomer A.C., Vaartjes I., van der Velde E.T., de Jong H.M.Y., Konings T.C., Wagenaar L.J., Heesen W.F., Eerens F., Baur L.H.B., Grobbee D.E. (2013). Heart Failure Admissions in Adults with Congenital Heart Disease; Risk Factors and Prognosis. Int. J. Cardiol..

[45] Wong C.M., Hawkins N.M., Ezekowitz J.A., Jhund P.S., Savu A., MacDonald M.R., Kristensen S.L., Petrie M.C., McMurray J.J.V., McAlister F.A. (2017). Heart Failure in Young Adults Is Associated with High Mortality: A Contemporary Population-Level Analysis. Can. J. Cardiol..

[46] van der Bom T., Winter M.M., Groenink M., Vliegen H.W., Pieper P.G., van Dijk A.P.J., Sieswerda G.T., Roos-Hesselink J.W., Zwinderman A.H., Mulder B.J.M. (2013). Right Ventricular End-Diastolic Volume Combined with Peak Systolic Blood Pressure During Exercise Identifies Patients at Risk for Complications in Adults with a Systemic Right Ventricle. J. Am. Coll. Cardiol..

[47] Lewis M.J., Van Dissel A., Kochav J., DiLorenzo M.P., Ginns J., Zemer-Wassercug N., Groenink M., Mulder B., Rosenbaum M. (2022). Cardiac MRI Predictors of Adverse Outcomes in Adults with a Systemic Right Ventricle. ESC Heart Fail..

[48] Winter M.M., Bernink F.J., Groenink M., Bouma B.J., van Dijk A.P., Helbing W.A., Tijssen J.G., Mulder B.J. (2008). Evaluating the Systemic Right Ventricle by CMR: The Importance of Consistent and Reproducible Delineation of the Cavity. J. Cardiovasc. Magn. Reson..

[49] Samyn M.M., Yan K., Masterson C., Goot B.H., Saudek D., Lavoie J., Kinney A., Krolikowski M., Hor K., Cohen S. (2019). Echocardiography *vs.* Cardiac Magnetic Resonance Imaging Assessment of the Systemic Right Ventricle for Patients with D-Transposition of the Great Arteries Status Post Atrial Switch. Congenit. Heart Dis..

[50] Geenen L.W., van Grootel R.W.J., Akman K., Baggen V.J.M., Menting M.E., Eindhoven J.A., Cuypers J.A.A.E., Boersma E., van den Bosch A.E., Roos-Hesselink J.W. (2019). Exploring the Prognostic Value of Novel Markers in Adults with a Systemic Right Ventricle. JAHA.

[51] Helsen F., De Meester P., Van De Bruaene A., Gabriels C., Santens B., Claeys M., Claessen G., Goetschalckx K., Buys R., Gewillig M. (2018). Right Ventricular Systolic Dysfunction at Rest Is Not Related to Decreased Exercise Capacity in Patients with a Systemic Right Ventricle. Int. J. Cardiol..

[52] Stauber A., Wey C., Greutmann M., Tobler D., Wustmann K., Wahl A., Valsangiacomo Buechel E.R., Wilhelm M., Schwerzmann M. (2017). Left Ventricular Outflow Tract Obstruction and Its Impact on Systolic Ventricular Function and Exercise Capacity in Adults with a Subaortic Right Ventricle. Int. J. Cardiol..

[53] Lebherz C., Gerhardus M., Lammers A.E., Helm P., Tutarel O., Bauer U., Bülow T., Kerst G., Diller G.-P., Marx N. (2022). Late Outcome, Therapy and Systemic Ventricular Function in Patients with a Systemic Right Ventricle: Data of the German National Register for Congenital Heart Defects. Cardiol. Young.

[54] Dubin A.M., Janousek J., Rhee E., Strieper M.J., Cecchin F., Law I.H., Shannon K.M., Temple J., Rosenthal E., Zimmerman F.J. (2005). Resynchronization Therapy in Pediatric and Congenital Heart Disease Patients: An International Multicenter Study. J. Am. Coll. Cardiol..

[55] Graham T.P., Parrish M.D., Boucek R.J., Boerth R.C., Breitweser J.A., Thompson S., Robertson R.M., Morgan J.R., Friesinger G.C. (1983). Assessment of Ventricular Size and Function in Congenitally Corrected Transposition of the Great Arteries. Am. J. Cardiol..

[56] Fredriksen P.M., Chen A., Veldtman G., Hechter S., Therrien J., Webb G. (2001). Exercise Capacity in Adult Patients with Congenitally Corrected Transposition of the Great Arteries. Heart.

[57] Lundstrom U., Bull C., Wyse R.K.H., Somerville J. (1990). The Natural and “Unnatural” History of Congenitally Corrected Transposition. Am. J. Cardiol..

[58] Dobson R., Danton M., Nicola W., Hamish W. (2013). The Natural and Unnatural History of the Systemic Right Ventricle in Adult Survivors. J. Thorac. Cardiovasc. Surg..

[59] Leeuwenburgh B.P., Helbing W.A., Steendijk P., Schoof P.H., Baan J. (2001). Biventricular Systolic Function in Young Lambs Subject to Chronic Systemic Right Ventricular Pressure Overload. Am. J. Physiol. Heart Circ. Physiol..

[60] Prieto L.R., Hordof A.J., Secic M., Rosenbaum M.S., Gersony W.M. (1998). Progressive Tricuspid Valve Disease in Patients with Congenitally Corrected Transposition of the Great Arteries. Circulation.

[61] Dos L. (2005). Late Outcome of Senning and Mustard Procedures for Correction of Transposition of the Great Arteries. Heart.

[62] Nartowicz S.A., Jakielska E., Ciepłucha A., Ratajczak P., Grajek S., Lesiak M., Trojnarska O. (2022). Clinical Factors Affecting Survival in Patients with D-Transposition of the Great Arteries after Atrial Switch Repair: A Meta-Analysis. Kardiol. Pol..

[63] Egbe A., Miranda W., Katta R.R., Goda A., Andi K., Connolly H. (2022). Determinants of Aerobic Capacity after Tricuspid Valve Replacement in Congenitally Corrected Transposition of Great Arteries. JACC Adv..

[64] Koolbergen D.R., Ahmed Y., Bouma B.J., Scherptong R.W.C., Bruggemans E.F., Vliegen H.W., Holman E.R., Mulder B.J.M., Hazekamp M.G. (2016). Follow-up after Tricuspid Valve Surgery in Adult Patients with Systemic Right Ventricles. Eur. J. Cardiothorac. Surg..

[65] Hernández-Madrid A., Paul T., Abrams D., Aziz P.F., Blom N.A., Chen J., Chessa M., Combes N., Dagres N., Diller G. (2018). Arrhythmias in Congenital Heart Disease: A Position Paper of the European Heart Rhythm Association (EHRA), Association for European Paediatric and Congenital Cardiology (AEPC), and the European Society of Cardiology (ESC) Working Group on Grown-up Congenital Heart Disease, Endorsed by HRS, PACES, APHRS, and SOLAECE. Europace.

[66] Baruteau A.-E., Abrams D.J., Ho S.Y., Thambo J.-B., McLeod C.J., Shah M.J. (2017). Cardiac Conduction System in Congenitally Corrected Transposition of the Great Arteries and Its Clinical Relevance. J. Am. Heart Assoc..

[67] Khairy P., Van Hare G.F., Balaji S., Berul C.I., Cecchin F., Cohen M.I., Daniels C.J., Deal B.J., Dearani J.A., De Groot N. (2014). PACES/HRS Expert Consensus Statement on the Recognition and Management of Arrhythmias in Adult Congenital Heart Disease: Developed in Partnership between the Pediatric and Congenital Electrophysiology Society (PACES) and the Heart Rhythm Society (HRS). Endorsed by the Governing Bodies of PACES, HRS, the American College of Cardiology (ACC), the American Heart Association (AHA), the European Heart Rhythm Association (EHRA), the Canadian Heart Rhythm Society (CHRS), and the International Society for Adult Congenital Heart Disease (ISACHD). Can. J. Cardiol..

[68] Gillette P.C., el-Said G.M., Sivarajan N., Mullins C.E., Williams R.L., McNamara D.G. (1974). Electrophysiological Abnormalities after Mustard’s Operation for Transposition of the Great Arteries. Br. Heart J..

[69] Jauvert G., Rousseau-Paziaud J., Villain E., Iserin L., Hidden-Lucet F., Ladouceur M., Sidi D. (2008). Effects of Cardiac Resynchronization Therapy on Echocardiographic Indices, Functional Capacity, and Clinical Outcomes of Patients with a Systemic Right Ventricle. Europace.

[70] Janousek J., Tomek V., Chaloupecký V., Reich O., Gebauer R.A., Kautzner J., Hucín B. (2004). Cardiac Resynchronization Therapy: A Novel Adjunct to the Treatment and Prevention of Systemic Right Ventricular Failure. J. Am. Coll. Cardiol..

[71] Diller G.-P., Okonko D., Uebing A., Ho S.Y., Gatzoulis M.A. (2006). Cardiac Resynchronization Therapy for Adult Congenital Heart Disease Patients with a Systemic Right Ventricle: Analysis of Feasibility and Review of Early Experience. EP Eur..

[72] Nederend M., Jongbloed M.R.M., Kiès P., Vliegen H.W., Bouma B.J., Regeer M.V., Koolbergen D.R., Hazekamp M.G., Schalij M.J., Egorova A.D. (2022). Atrioventricular Block Necessitating Chronic Ventricular Pacing after Tricuspid Valve Surgery in Patients with a Systemic Right Ventricle: Long-Term Follow-Up. Front. Cardiovasc. Med..

[73] Nederend M., van Erven L., Zeppenfeld K., Vliegen H.W., Egorova A.D. (2021). Failing Systemic Right Ventricle in a Patient with Dextrocardia and Complex Congenitally Corrected Transposition of the Great Arteries: A Case Report of Successful Transvenous Cardiac Resynchronization Therapy. Eur. Heart J. Case Rep..

[74] Bottega N.A., Kapa S., Edwards W.D., Connolly H.M., Munger T.M., Warnes C.A., Asirvatham S.J. (2009). The Cardiac Veins in Congenitally Corrected Transposition of the Great Arteries: Delivery Options for Cardiac Devices. Heart Rhythm.

[75] Moore J.P., Cho D., Lin J.P., Lluri G., Reardon L.C., Aboulhosn J.A., Hageman A., Shannon K.M. (2018). Implantation Techniques and Outcomes after Cardiac Resynchronization Therapy for Congenitally Corrected Transposition of the Great Arteries. Heart Rhythm.

[76] Yeo W.T., Jarman J.W.E., Li W., Gatzoulis M.A., Wong T. (2014). Adverse Impact of Chronic Subpulmonary Left Ventricular Pacing on Systemic Right Ventricular Function in Patients with Congenitally Corrected Transposition of the Great Arteries. Int. J. Cardiol..

[77] Kharbanda R.K., Moore J.P., Lloyd M.S., Galotti R., Bogers A.J.J.C., Taverne Y.J.H.J., Madhavan M., McLeod C.J., Dubin A.M., Mah D.Y. (2022). Cardiac Resynchronization Therapy for Adult Patients with a Failing Systemic Right Ventricle: A Multicenter Study. J. Am. Heart Assoc..

[78] Haberger S., Hauser M., Braun S.L., Schuster T., Ewert P., Nagdyman N., Hess J., Kaemmerer H. (2015). Prognostic Value of Plasma B-Type Natriuretic Peptide in the Long-Term Follow-up of Patients with Transposition of the Great Arteries with Morphologic Right Systemic Ventricle after Atrial Switch Operation. Circ. J..

[79] Diller G.-P., Kempny A., Alonso-Gonzalez R., Swan L., Uebing A., Li W., Babu-Narayan S., Wort S.J., Dimopoulos K., Gatzoulis M.A. (2015). Survival Prospects and Circumstances of Death in Contemporary Adult Congenital Heart Disease Patients under Follow-Up at a Large Tertiary Centre. Circulation.

[80] Popelová J.R., Kotaška K., Tomková M., Tomek J. (2015). Usefulness of N-Terminal Pro-Brain Natriuretic Peptide to Predict Mortality in Adults with Congenital Heart Disease. Am. J. Cardiol..

[81] Westhoff-Bleck M., Podewski E., Tutarel O., Wenzel D., Cappello C., Bertram H., Bauersachs J., Widder J. (2013). Prognostic Value of NT-ProBNP in Patients with Systemic Morphological Right Ventricles: A Single-Centre Experience. Int. J. Cardiol..

[82] Aarsvold K.J., Danford D.A., Yetman A.T. (2022). Oxygen Uptake Efficiency Slope Predicts Adverse Outcome Following Atrial Switch Procedure. Pediatr. Cardiol..

[83] Ladouceur M., Kachenoura N., Soulat G., Bollache E., Redheuil A., Azizi M., Delclaux C., Chatellier G., Boutouyrie P., Iserin L. (2017). Impaired Atrioventricular Transport in Patients with Transposition of the Great Arteries Palliated by Atrial Switch and Preserved Systolic Right Ventricular Function: A Magnetic Resonance Imaging Study: LADOUCEUR et al. Congenit. Heart Dis..

[84] Holland R., Rechel B., Stepien K., Harvey I., Brooksby I. (2010). Patients’ Self-Assessed Functional Status in Heart Failure by New York Heart Association Class: A Prognostic Predictor of Hospitalizations, Quality of Life and Death. J. Card Fail..

[85] Das B.B., Godoy A., Kadish T., Niu J. (2021). Maximal versus Sub-Maximal Effort during Cardiopulmonary Exercise Testing in Adults with Congenital Heart Disease: Outcome Analysis of Short-Term Cardiac-Related Events. Cardiol. Young.

[86] Giardini A., Hager A., Lammers A.E., Derrick G., Müller J., Diller G.-P., Dimopoulos K., Odendaal D., Gargiulo G., Picchio F.M. (2009). Ventilatory Efficiency and Aerobic Capacity Predict Event-Free Survival in Adults with Atrial Repair for Complete Transposition of the Great Arteries. J. Am. Coll. Cardiol..

[87] Eindhoven J.A., van den Bosch A.E., Jansen P.R., Boersma E., Roos-Hesselink J.W. (2012). The Usefulness of Brain Natriuretic Peptide in Complex Congenital Heart Disease: A Systematic Review. J. Am. Coll. Cardiol..

[88] Popelová J.R., Tomková M., Tomek J. (2017). NT-ProBNP Predicts Mortality in Adults with Transposition of the Great Arteries Late after Mustard or Senning Correction: POPELOVÁ et al. Congenit. Heart Dis..

[89] Koch A.M.E., Zink S., Singer H. (2008). B-Type Natriuretic Peptide in Patients with Systemic Right Ventricle. Cardiology.

[90] Plymen C.M., Hughes M.L., Picaut N., Panoulas V.F., MacDonald S.T., Cullen S., Deanfield J.E., Walker F., Taylor A.M., Lambiase P.D. (2010). The Relationship of Systemic Right Ventricular Function to ECG Parameters and NT-ProBNP Levels in Adults with Transposition of the Great Arteries Late after Senning or Mustard Surgery. Heart.

[91] Schaefer A., Tallone E.M., Westhoff-Bleck M., Klein G., Drexler H., Röntgen P. (2010). Relation of Diastolic and Systolic Function, Exercise Capacity and Brain Natriuretic Peptide in Adults after Mustard Procedure for Transposition of the Great Arteries. Cardiology.

[92] Abu-Halima M., Meese E., Abdul-Khaliq H., Raedle-Hurst T. (2021). MicroRNA-183-3p Is a Predictor of Worsening Heart Failure in Adult Patients with Transposition of the Great Arteries and a Systemic Right Ventricle. Front. Cardiovasc. Med..

[93] Van De Bruaene A., Hickey E.J., Kovacs A.H., Crean A.M., Wald R.M., Silversides C.K., Redington A.N., Ross H.J., Alba A.C., Billia F. (2018). Phenotype, Management and Predictors of Outcome in a Large Cohort of Adult Congenital Heart Disease Patients with Heart Failure. Int. J. Cardiol..

[94] Zaragoza-Macias E., Zaidi A.N., Dendukuri N., Marelli A. (2019). Medical Therapy for Systemic Right Ventricles: A Systematic Review (Part 1) for the 2018 AHA/ACC Guideline for the Management of Adults with Congenital Heart Disease: A Report of the American College of Cardiology/American Heart Association Task Force on Clinical Practice Guidelines. Circulation.

[95] van der Bom T., Winter M.M., Bouma B.J., Groenink M., Vliegen H.W., Pieper P.G., van Dijk A.P.J., Sieswerda G.T., Roos-Hesselink J.W., Zwinderman A.H. (2013). Effect of Valsartan on Systemic Right Ventricular Function: A Double-Blind, Randomized, Placebo-Controlled Pilot Trial. Circulation.

[96] Zandstra T.E., Nederend M., Jongbloed M.R.M., Kiès P., Vliegen H.W., Bouma B.J., Tops L.F., Schalij M.J., Egorova A.D. (2021). Sacubitril/Valsartan in the Treatment of Systemic Right Ventricular Failure. Heart.

[97] van Son J.A., Reddy V.M., Silverman N.H., Hanley F.L. (1996). Regression of Tricuspid Regurgitation after Two-Stage Arterial Switch Operation for Failing Systemic Ventricle after Atrial Inversion Operation. J. Thorac. Cardiovasc. Surg..

[98] Kral Kollars C.A., Gelehrter S., Bove E.L., Ensing G. (2010). Effects of Morphologic Left Ventricular Pressure on Right Ventricular Geometry and Tricuspid Valve Regurgitation in Patients with Congenitally Corrected Transposition of the Great Arteries. Am. J. Cardiol..

[99] Winlaw D.S., McGuirk S.P., Balmer C., Langley S.M., Griselli M., Stümper O., De Giovanni J.V., Wright J.G., Thorne S., Barron D.J. (2005). Intention-to-Treat Analysis of Pulmonary Artery Banding in Conditions with a Morphological Right Ventricle in the Systemic Circulation with a View to Anatomic Biventricular Repair. Circulation.

[100] Poirier N.C., Yu J.-H., Brizard C.P., Mee R.B.B. (2004). Long-Term Results of Left Ventricular Reconditioning and Anatomic Correction for Systemic Right Ventricular Dysfunction after Atrial Switch Procedures. J. Thorac. Cardiovasc. Surg..

[101] Hörer J., Herrmann F., Schreiber C., Cleuziou J., Prodan Z., Vogt M., Holper K., Lange R. (2007). How Well Are Patients Doing up to 30 Years after a Mustard Operation?. Thorac. Cardiovasc. Surg..

[102] Singh T.P., Humes R.A., Muzik O., Kottamasu S., Karpawich P.P., Di Carli M.F. (2001). Myocardial Flow Reserve in Patients with a Systemic Right Ventricle after Atrial Switch Repair. J. Am. Coll. Cardiol..

[103] Ross H.J., Law Y., Book W.M., Broberg C.S., Burchill L., Cecchin F., Chen J.M., Delgado D., Dimopoulos K., Everitt M.D. (2016). Transplantation and Mechanical Circulatory Support in Congenital Heart Disease. Circulation.

[104] Davies R.R., Russo M.J., Yang J., Quaegebeur J.M., Mosca R.S., Chen J.M. (2011). Listing and Transplanting Adults with Congenital Heart Disease. Circulation.

[105] Gyoten T., Rojas S.V., Fox H., Schramm R., Hakim-Meibodi K., Ruiz-Cano M., Gummert J.F., Morshuis M., Sandica E. (2021). Mechanical Circulatory Support as a Bridge to Candidacy in Adults with Transposition of the Great Arteries and a Systemic Right Ventricle. Eur. J. Cardio-Thorac. Surg..

[106] Peng E., O’Sullivan J.J., Griselli M., Roysam C., Crossland D., Chaudhari M., Wrightson N., Butt T., Parry G., MacGowan G.A. (2014). Durable Ventricular Assist Device Support for Failing Systemic Morphologic Right Ventricle: Early Results. Ann. Thorac. Surg..

[107] Zandstra T.E., Palmen M., Hazekamp M.G., Meyns B., Beeres S.L.M.A., Holman E.R., Kiès P., Jongbloed M.R.M., Vliegen H.W., Egorova A.D. (2019). Ventricular Assist Device Implantation in Patients with a Failing Systemic Right Ventricle: A Call to Expand Current Practice. Neth. Heart J..

[108] Shah D.K., Deo S.V., Althouse A.D., Teuteberg J.J., Park S.J., Kormos R.L., Burkhart H.M., Morell V.O. (2016). Perioperative Mortality Is the Achilles Heel for Cardiac Transplantation in Adults with Congenital Heart Disease: Evidence from Analysis of the UNOS Registry. J. Card. Surg..

[109] Monaco J., Khanna A., Khazanie P. (2020). Transplant and Mechanical Circulatory Support in Patients with Adult Congenital Heart Disease. Heart Fail. Rev..

[110] Koolbergen D.R. (2022). Mechanical Circulatory Support in the Failing Systemic Right Ventricle: A Step towards Better Outcome. Eur. J. Cardio-Thorac. Surg..

[111] Regitz-Zagrosek V., Roos-Hesselink J.W., Bauersachs J., Blomström-Lundqvist C., Cífková R., De Bonis M., Iung B., Johnson M.R., Kintscher U., Kranke P. (2018). 2018 ESC Guidelines for the Management of Cardiovascular Diseases during Pregnancy. Eur. Heart J..

[112] Drenthen W., Pieper P.G., Roos-Hesselink J.W., van Lottum W.A., Voors A.A., Mulder B.J.M., van Dijk A.P.J., Vliegen H.W., Yap S.C., Moons P. (2007). Outcome of Pregnancy in Women with Congenital Heart Disease: A Literature Review. J. Am. Coll. Cardiol..

[113] Drenthen W., Pieper P.G., Ploeg M., Voors A.A., Roos-Hesselink J.W., Mulder B.J.M., Vliegen H.W., Sollie K.M., Ebels T., van Veldhuisen D.J. (2005). Risk of Complications during Pregnancy after Senning or Mustard (Atrial) Repair of Complete Transposition of the Great Arteries. Eur. Heart J..

[114] Canobbio M.M., Morris C.D., Graham T.P., Landzberg M.J. (2006). Pregnancy Outcomes after Atrial Repair for Transposition of the Great Arteries. Am. J. Cardiol..

[115] Trigas V., Nagdyman N., Pildner von Steinburg S., Oechslin E., Vogt M., Berger F., Schneider K.-T.M., Ewert P., Hess J., Kaemmerer H. (2014). Pregnancy-Related Obstetric and Cardiologic Problems in Women after Atrial Switch Operation for Transposition of the Great Arteries. Circ. J..

[116] Tutarel O., Baris L., Budts W., Gamal Abd-El Aziz M., Liptai C., Majdalany D., Jovanova S., Frogoudaki A., Connolly H.M., Johnson M.R. (2022). Pregnancy Outcomes in Women with a Systemic Right Ventricle and Transposition of the Great Arteries Results from the ESC-EORP Registry of Pregnancy and Cardiac Disease (ROPAC). Heart.

[117] Cataldo S., Doohan M., Rice K., Trinder J., Stuart A., Curtis S. (2016). Pregnancy Following Mustard or Senning Correction of Transposition of the Great Arteries: A Retrospective Study. BJOG: Int. J. Obstet. Gynaecol..

[118] Khairy P., Silka M.J., Moore J.P., DiNardo J.A., Vehmeijer J.T., Sheppard M.N., van de Bruaene A., Chaix M.-A., Brida M., Moore B.M. (2022). Sudden Cardiac Death in Congenital Heart Disease. Eur. Heart J..

[119] Ladouceur M., Van De Bruaene A., Kauling R., Budts W., Roos-Hesselink J., Albert S.V., Perez I.S., Sarubbi B., Fusco F., Gallego P. (2022). A New Score for Life-Threatening Ventricular Arrhythmias and Sudden Cardiac Death in Adults with Transposition of the Great Arteries and a Systemic Right Ventricle. Eur. Heart J..

[120] Jensen A.S., Jørgensen T.H., Christersson C., Nagy E., Sinisalo J., Furenäs E., Gjesdal O., Eriksson P., Vejlstrup N., Johansson B. (2022). Cause-Specific Mortality in Patients During Long-Term Follow-Up after Atrial Switch for Transposition of the Great Arteries. J. Am. Heart Assoc..

[121] Broberg C.S., van Dissel A., Minnier J., Aboulhosn J., Kauling R.M., Ginde S., Krieger E.V., Rodriguez F., Gupta T., Shah S. (2022). Long-Term Outcomes after Atrial Switch Operation for Transposition of the Great Arteries. J. Am. Coll. Cardiol..

[122] Love B.A., Mehta D., Fuster V.F. (2008). Evaluation and Management of the Adult Patient with Transposition of the Great Arteries Following Atrial-Level (Senning or Mustard) Repair. Nat. Rev. Cardiol..

[123] Kammeraad J.A.E., van Deurzen C.H.M., Sreeram N., Bink-Boelkens M.T.E., Ottenkamp J., Helbing W.A., Lam J., Sobotka-Plojhar M.A., Daniels O., Balaji S. (2004). Predictors of Sudden Cardiac Death after Mustard or Senning Repair for Transposition of the Great Arteries. J. Am. Coll. Cardiol..

[124] Khairy P., Harris L., Landzberg M.J., Fernandes S.M., Barlow A., Mercier L.-A., Viswanathan S., Chetaille P., Gordon E., Dore A. (2008). Sudden Death and Defibrillators in Transposition of the Great Arteries with Intra-Atrial Baffles: A Multicenter Study. Circ. Arrhythmia Electrophysiol..

[125] Khairy P. (2017). Sudden Cardiac Death in Transposition of the Great Arteries with a Mustard or Senning Baffle: The Myocardial Ischemia Hypothesis. Curr. Opin. Cardiol..

[126] Kapa S., Vaidya V., Hodge D.O., McLeod C.J., Connolly H.M., Warnes C.A., Asirvatham S.J. (2018). Right Ventricular Dysfunction in Congenitally Corrected Transposition of the Great Arteries and Risk of Ventricular Tachyarrhythmia and Sudden Death. Int. J. Cardiol..

[127] Daliento L., Corrado D., Buja G., John N., Nava A., Thiene G. (1986). Rhythm and Conduction Disturbances in Isolated, Congenitally Corrected Transposition of the Great Arteries. Am. J. Cardiol..

[128] Buber J., Ackley T.J., Daniels C.J., Roble S.L., Mah M.L., Kamp A.N., Kertesz N.J. (2016). Outcomes Following the Implantation of Cardioverter-Defibrillator for Primary Prevention in Transposition of the Great Arteries after Intra-Atrial Baffle Repair: A Single-Centre Experience. Europace.

[129] Bouzeman A., Marijon E., de Guillebon M., Ladouceur M., Duthoit G., Amet D., Martins R., Otmani A., Lavergne T., Bordachar P. (2014). Implantable Cardiac Defibrillator among Adults with Transposition of the Great Arteries and Atrial Switch Operation: Case Series and Review of Literature. Int. J. Cardiol..

[130] Ellenbogen K.A., Levine J.H., Berger R.D., Daubert J.P., Winters S.L., Greenstein E., Shalaby A., Schaechter A., Subacius H., Kadish A. (2006). Are Implantable Cardioverter Defibrillator Shocks a Surrogate for Sudden Cardiac Death in Patients with Nonischemic Cardiomyopathy?. Circulation.

[131] Silka M.J., Bar-Cohen Y. (2008). Should Patients with Congenital Heart Disease and a Systemic Ventricular Ejection Fraction Less than 30% undergo Prophylactic Implantation of an ICD? Patients with Congenital Heart Disease and a Systemic Ventricular Ejection Fraction Less than 30% Should undergo Prophylactic Implantation of an Implantable Cardioverter Defibrillator. Circ. Arrhythm. Electrophysiol..

[132] Helsen F., De Meester P., Van Keer J., Gabriels C., Van De Bruaene A., Herijgers P., Rega F., Meyns B., Gewillig M., Troost E. (2015). Pulmonary Outflow Obstruction Protects against Heart Failure in Adults with Congenitally Corrected Transposition of the Great Arteries. Int. J. Cardiol..

[133] Woudstra O.I., Skoric-Milosavljevic D., Mulder B.J.M., Meijboom F.J., Post M.C., Jongbloed M.R.M., van Dijk A.P.J., van Melle J.P., Konings T.C., Postma A.V. (2022). Common Genetic Variants Improve Risk Stratification after the Atrial Switch Operation for Transposition of the Great Arteries. Int. J. Cardiol..

